# Supplementary material for: Parallel evolution of gene expression between trophic specialists despite divergent genotypes and morphologies
Source: Evol Lett. 2018 Feb 14;2(2):62–75. doi: 10.1002/evl3.41 (PMC6089502; doi:10.1002/evl3.41)
Supplement: Supplementary file 1 — Table S1. Total mRNA sequencing sampling design. Table S2. Four genes showing opposite expression patterns in specialists relative to generalists. Table S3. Enriched gene ontologies for genes showing parallel changes in expression between specialists. Table S4. Enriched gene ontologies for genes showing divergent expression in specialists. Table S5. Eleven genes previously described as candidates influencing craniofacial divergence are differentially expressed between generalists and specialists (McGirr and Martin 2017). Table S6. 68 out of 84 gene regions containing fixed variants show signs of a hard sweep (estimated by SweeD; CLR > 95th percentile across their respective scaffolds). Fig. S1. A similar number of reads map to annotated features across generalists (red), snail‐eaters (green), and scale‐eaters (blue) (ANOVA; 8–10 dpf P = 0.47; 17–20 dpf P = 0.33). Fig. S2. Null distributions of parallel changes in gene expression between specialists. Fig. S3. Parallel changes in isoform expression between specialists at 8–10 dpf. Fig. S4. Significant parallel evolution of gene expression between specialists despite divergent trophic adaptation. Fig. S5. Down sampling permutations. Distribution of genes differentially expressed (DE) between generalists and snail‐eaters (A and B), generalists and scale‐eaters (C, and D), and genes DE in both comparisons (E and F) for 8–10 dpf (left) and 17–20 dpf (right) samples after 1000 down sampling permutations where groups of generalists and snail‐eaters were randomly sampled to match scale‐eater sample sizes (8–10 dpf, n = 3; 17–20 dpf, n = 2). Fig. S6. Genes showing parallel expression patterns in specialists are not more pleiotropic than genes showing divergent expression. Fig. S7. Fst permutations to determine significantly differentiated SNPs. [file EVL3-2-62-s001.docx]

**Table S1.** Total mRNA sequencing sampling design.

| species | 8-10 dpf | 17-20 dpf | 8-10 dpf | 17-20 dpf |
| --- | --- | --- | --- | --- |
| generalist | 3 F_2_ | 3 F_2_ | 3 F_1_ | 3 F_1_ |
| snail-eater | 3 F_2_ | 3 F_2_ | 3 F_2_ | 3 F_2_ |
| scale-eater | 0 | 0 | 3 F_1_ | 2 F_1_ |

Crescent Pond Little Lake

**Table S2.** Four genes showing opposite expression patterns in specialists relative to generalists.

snail-eater scale-eater

*vs. vs.*

generalist generalist

| scaffold | stage | symbol | zebrafish ortholog | log_2_ FC | *P* | log_2_ FC | *P* |
| --- | --- | --- | --- | --- | --- | --- | --- |
| 015150477 | 17-20 dpf | LOC107096735 | mybpc2a | 2.13 | 0.05 | -3.27 | 0.00 |
| 015150518 | 8-10 dpf | LOC107082892 | si:ch211-197h24.9 | 2.90 | 0.01 | -2.61 | 0.02 |
| 015150587 | 8-10 dpf | agxt2 | agxt2 | 1.48 | 0.01 | -1.43 | 0.01 |
| 015150546 | 8-10 dpf | plin2 | plin2 | -0.90 | 0.03 | 2.43 | 0.00 |

**Table S3.** Enriched gene ontologies for genes showing parallel changes in expression between specialists. Representative terms were determined using REVIGO (Tomislav 2011).

| ID | description | representative term |
| --- | --- | --- |
| GO:0006950 | response to stress | response to stress |
| GO:0006302 | double-strand break repair | response to stress |
| GO:0006974 | cellular response to DNA damage stimulus | response to stress |
| GO:0000725 | recombinational repair | response to stress |
| GO:0000724 | double-strand break repair via homologous recombination | response to stress |
| GO:0008150 | biological_process | biological_process |
| GO:0008152 | metabolic process | metabolism |
| GO:0009987 | cellular process | cellular process |
| GO:0032501 | multicellular organismal process | multicellular organismal process |
| GO:0032502 | developmental process | developmental process |
| GO:0044085 | cellular component biogenesis | cellular component biogenesis |
| GO:0048285 | organelle fission | cellular component biogenesis |
| GO:0000280 | nuclear division | cellular component biogenesis |
| GO:0071826 | ribonucleoprotein complex subunit organization | cellular component biogenesis |
| GO:0043933 | macromolecular complex subunit organization | cellular component biogenesis |
| GO:0033043 | regulation of organelle organization | cellular component biogenesis |
| GO:0042254 | ribosome biogenesis | cellular component biogenesis |
| GO:0070925 | organelle assembly | cellular component biogenesis |
| GO:0016570 | histone modification | cellular component biogenesis |
| GO:0006325 | chromatin organization | cellular component biogenesis |
| GO:0007010 | cytoskeleton organization | cellular component biogenesis |
| GO:0006996 | organelle organization | cellular component biogenesis |
| GO:0051128 | regulation of cellular component organization | cellular component biogenesis |
| GO:0030261 | chromosome condensation | cellular component biogenesis |
| GO:0044699 | single-organism process | single-organism process |
| GO:0044707 | single-multicellular organism process | single-multicellular organism process |
| GO:0044710 | single-organism metabolic process | single-multicellular organism process |
| GO:0044763 | single-organism cellular process | single-multicellular organism process |
| GO:0044281 | small molecule metabolic process | single-multicellular organism process |
| GO:0060041 | retina development in camera-type eye | single-multicellular organism process |
| GO:0048519 | negative regulation of biological process | negative regulation of biological process |
| GO:0080090 | regulation of primary metabolic process | negative regulation of biological process |
| GO:0031324 | negative regulation of cellular metabolic process | negative regulation of biological process |
| GO:0019222 | regulation of metabolic process | negative regulation of biological process |
| GO:0050794 | regulation of cellular process | negative regulation of biological process |
| GO:0048856 | anatomical structure development | anatomical structure development |
| GO:0051303 | establishment of chromosome localization | establishment of chromosome localization |
| GO:0065007 | biological regulation | biological regulation |
| GO:0071840 | cellular component organization or biogenesis | cellular component organization or biogenesis |
| GO:1901575 | organic substance catabolic process | organic substance catabolism |
| GO:0009056 | catabolic process | catabolism |
| GO:0009058 | biosynthetic process | biosynthesis |
| GO:0006479 | protein methylation | protein methylation |
| GO:0090304 | nucleic acid metabolic process | protein methylation |
| GO:0055086 | nucleobase-containing small molecule metabolic process | protein methylation |
| GO:0019538 | protein metabolic process | protein methylation |
| GO:0006310 | DNA recombination | protein methylation |
| GO:0002181 | cytoplasmic translation | protein methylation |
| GO:0006518 | peptide metabolic process | protein methylation |
| GO:0006271 | DNA strand elongation involved in DNA replication | protein methylation |
| GO:0010467 | gene expression | protein methylation |
| GO:0006275 | regulation of DNA replication | protein methylation |
| GO:0006261 | DNA-dependent DNA replication | protein methylation |
| GO:0018205 | peptidyl-lysine modification | protein methylation |
| GO:0022616 | DNA strand elongation | protein methylation |
| GO:0019752 | carboxylic acid metabolic process | protein methylation |
| GO:0016070 | RNA metabolic process | protein methylation |
| GO:0016072 | rRNA metabolic process | protein methylation |
| GO:0044260 | cellular macromolecule metabolic process | protein methylation |
| GO:0043412 | macromolecule modification | protein methylation |
| GO:0051052 | regulation of DNA metabolic process | protein methylation |
| GO:0006082 | organic acid metabolic process | protein methylation |
| GO:0008213 | protein alkylation | protein methylation |
| GO:0044267 | cellular protein metabolic process | protein methylation |
| GO:0006413 | translational initiation | protein methylation |
| GO:1901564 | organonitrogen compound metabolic process | protein methylation |
| GO:1901566 | organonitrogen compound biosynthetic process | protein methylation |
| GO:0006139 | nucleobase-containing compound metabolic process | protein methylation |
| GO:0043603 | cellular amide metabolic process | protein methylation |
| GO:0072521 | purine-containing compound metabolic process | protein methylation |
| GO:0045005 | DNA-dependent DNA replication maintenance of fidelity | protein methylation |
| GO:0034641 | cellular nitrogen compound metabolic process | protein methylation |
| GO:0006464 | cellular protein modification process | protein methylation |
| GO:0006259 | DNA metabolic process | protein methylation |
| GO:0006260 | DNA replication | protein methylation |
| GO:0043038 | amino acid activation | protein methylation |
| GO:0034660 | ncRNA metabolic process | protein methylation |
| GO:0006807 | nitrogen compound metabolic process | nitrogen compound metabolism |
| GO:0019693 | ribose phosphate metabolic process | ribose phosphate metabolism |
| GO:0019637 | organophosphate metabolic process | ribose phosphate metabolism |
| GO:0009141 | nucleoside triphosphate metabolic process | ribose phosphate metabolism |
| GO:1901135 | carbohydrate derivative metabolic process | carbohydrate derivative metabolism |
| GO:0044711 | single-organism biosynthetic process | carbohydrate derivative metabolism |
| GO:0009059 | macromolecule biosynthetic process | carbohydrate derivative metabolism |
| GO:0044249 | cellular biosynthetic process | carbohydrate derivative metabolism |
| GO:0043170 | macromolecule metabolic process | carbohydrate derivative metabolism |
| GO:0044271 | cellular nitrogen compound biosynthetic process | carbohydrate derivative metabolism |
| GO:1901576 | organic substance biosynthetic process | carbohydrate derivative metabolism |
| GO:1901362 | organic cyclic compound biosynthetic process | carbohydrate derivative metabolism |
| GO:1901360 | organic cyclic compound metabolic process | carbohydrate derivative metabolism |
| GO:0034645 | cellular macromolecule biosynthetic process | carbohydrate derivative metabolism |
| GO:0007059 | chromosome segregation | chromosome segregation |
| GO:0000278 | mitotic cell cycle | chromosome segregation |
| GO:0061640 | cytoskeleton-dependent cytokinesis | chromosome segregation |
| GO:0007049 | cell cycle | chromosome segregation |
| GO:0051301 | cell division | chromosome segregation |
| GO:0007017 | microtubule-based process | chromosome segregation |
| GO:0007018 | microtubule-based movement | chromosome segregation |
| GO:0045787 | positive regulation of cell cycle | chromosome segregation |
| GO:0044238 | primary metabolic process | primary metabolism |
| GO:0006793 | phosphorus metabolic process | primary metabolism |
| GO:0044237 | cellular metabolic process | primary metabolism |
| GO:0046483 | heterocycle metabolic process | primary metabolism |
| GO:0071704 | organic substance metabolic process | primary metabolism |
| GO:0006725 | cellular aromatic compound metabolic process | primary metabolism |

**Table S4.** Enriched gene ontologies for genes showing divergent expression in specialists. Representative terms were determined using REVIGO (Tomislav 2011).

| ID | description | representative term |
| --- | --- | --- |
| GO:0002376 | immune system process | immune system process |
| GO:0006839 | mitochondrial transport | mitochondrial transport |
| GO:0015031 | protein transport | mitochondrial transport |
| GO:0006820 | anion transport | mitochondrial transport |
| GO:0071705 | nitrogen compound transport | mitochondrial transport |
| GO:0034504 | protein localization to nucleus | mitochondrial transport |
| GO:0016192 | vesicle-mediated transport | mitochondrial transport |
| GO:0015931 | nucleobase-containing compound transport | mitochondrial transport |
| GO:0050658 | RNA transport | mitochondrial transport |
| GO:0000041 | transition metal ion transport | mitochondrial transport |
| GO:0033036 | macromolecule localization | mitochondrial transport |
| GO:0042886 | amide transport | mitochondrial transport |
| GO:0015833 | peptide transport | mitochondrial transport |
| GO:0006403 | RNA localization | mitochondrial transport |
| GO:1990542 | mitochondrial transmembrane transport | mitochondrial transport |
| GO:0048193 | Golgi vesicle transport | mitochondrial transport |
| GO:0051641 | cellular localization | mitochondrial transport |
| GO:0015711 | organic anion transport | mitochondrial transport |
| GO:0009620 | response to fungus | response to fungus |
| GO:1901698 | response to nitrogen compound | response to fungus |
| GO:0034976 | response to endoplasmic reticulum stress | response to fungus |
| GO:0048583 | regulation of response to stimulus | response to fungus |
| GO:0036503 | ERAD pathway | response to fungus |
| GO:0009628 | response to abiotic stimulus | response to fungus |
| GO:0009605 | response to external stimulus | response to fungus |
| GO:0042221 | response to chemical | response to fungus |
| GO:0010033 | response to organic substance | response to fungus |
| GO:0006974 | cellular response to DNA damage stimulus | response to fungus |
| GO:0010243 | response to organonitrogen compound | response to fungus |
| GO:0032259 | methylation | methylation |
| GO:0032501 | multicellular organismal process | multicellular organismal process |
| GO:0032502 | developmental process | developmental process |
| GO:0040007 | growth | growth |
| GO:0050790 | regulation of catalytic activity | regulation of catalytic activity |
| GO:0048519 | negative regulation of biological process | regulation of catalytic activity |
| GO:0048518 | positive regulation of biological process | regulation of catalytic activity |
| GO:0023051 | regulation of signaling | regulation of catalytic activity |
| GO:0051246 | regulation of protein metabolic process | regulation of catalytic activity |
| GO:0006357 | regulation of transcription from RNA polymerase II promoter | regulation of catalytic activity |
| GO:0035556 | intracellular signal transduction | regulation of catalytic activity |
| GO:0042592 | homeostatic process | regulation of catalytic activity |
| GO:0010646 | regulation of cell communication | regulation of catalytic activity |
| GO:0007267 | cell-cell signaling | regulation of catalytic activity |
| GO:0010608 | posttranscriptional regulation of gene expression | regulation of catalytic activity |
| GO:0065009 | regulation of molecular function | regulation of catalytic activity |
| GO:0065008 | regulation of biological quality | regulation of catalytic activity |
| GO:0007186 | G-protein coupled receptor signaling pathway | regulation of catalytic activity |
| GO:0034101 | erythrocyte homeostasis | regulation of catalytic activity |
| GO:0048872 | homeostasis of number of cells | regulation of catalytic activity |
| GO:0009893 | positive regulation of metabolic process | regulation of catalytic activity |
| GO:0009892 | negative regulation of metabolic process | regulation of catalytic activity |
| GO:0034248 | regulation of cellular amide metabolic process | regulation of catalytic activity |
| GO:0034249 | negative regulation of cellular amide metabolic process | regulation of catalytic activity |
| GO:0061077 | chaperone-mediated protein folding | chaperone-mediated protein folding |
| GO:1904888 | cranial skeletal system development | cranial skeletal system development |
| GO:0048589 | developmental growth | cranial skeletal system development |
| GO:0061061 | muscle structure development | cranial skeletal system development |
| GO:0055002 | striated muscle cell development | cranial skeletal system development |
| GO:0010927 | cellular component assembly involved in morphogenesis | cranial skeletal system development |
| GO:0048863 | stem cell differentiation | cranial skeletal system development |
| GO:0048856 | anatomical structure development | cranial skeletal system development |
| GO:0002072 | optic cup morphogenesis involved in camera-type eye development | cranial skeletal system development |
| GO:0014706 | striated muscle tissue development | cranial skeletal system development |
| GO:0060322 | head development | cranial skeletal system development |
| GO:0048705 | skeletal system morphogenesis | cranial skeletal system development |
| GO:0046148 | pigment biosynthetic process | pigment biosynthesis |
| GO:0044723 | single-organism carbohydrate metabolic process | pigment biosynthesis |
| GO:0006629 | lipid metabolic process | pigment biosynthesis |
| GO:0042440 | pigment metabolic process | pigment biosynthesis |
| GO:1901615 | organic hydroxy compound metabolic process | organic hydroxy compound metabolism |
| GO:0006914 | autophagy | autophagy |
| GO:0016236 | macroautophagy | macroautophagy |
| GO:0007033 | vacuole organization | vacuole organization |
| GO:0071826 | ribonucleoprotein complex subunit organization | vacuole organization |
| GO:0044802 | single-organism membrane organization | vacuole organization |
| GO:0006325 | chromatin organization | vacuole organization |
| GO:0097435 | supramolecular fiber organization | vacuole organization |
| GO:0044085 | cellular component biogenesis | vacuole organization |
| GO:0044087 | regulation of cellular component biogenesis | vacuole organization |
| GO:0000469 | cleavage involved in rRNA processing | vacuole organization |
| GO:0000466 | maturation of 5.8S rRNA from tricistronic rRNA transcript (SSU-rRNA, 5.8S rRNA, LSU-rRNA) | vacuole organization |
| GO:0043933 | macromolecular complex subunit organization | vacuole organization |
| GO:0042254 | ribosome biogenesis | vacuole organization |
| GO:0070925 | organelle assembly | vacuole organization |
| GO:0016570 | histone modification | vacuole organization |
| GO:0007010 | cytoskeleton organization | vacuole organization |
| GO:0007005 | mitochondrion organization | vacuole organization |
| GO:0006996 | organelle organization | vacuole organization |
| GO:0030036 | actin cytoskeleton organization | vacuole organization |
| GO:0051128 | regulation of cellular component organization | vacuole organization |
| GO:0043254 | regulation of protein complex assembly | vacuole organization |
| GO:0006457 | protein folding | protein folding |
| GO:0006790 | sulfur compound metabolic process | sulfur compound metabolism |
| GO:1901137 | carbohydrate derivative biosynthetic process | carbohydrate derivative biosynthesis |
| GO:0009100 | glycoprotein metabolic process | carbohydrate derivative biosynthesis |
| GO:0009101 | glycoprotein biosynthetic process | carbohydrate derivative biosynthesis |
| GO:0033013 | tetrapyrrole metabolic process | tetrapyrrole metabolism |
| GO:0016072 | rRNA metabolic process | tetrapyrrole metabolism |
| GO:0016071 | mRNA metabolic process | tetrapyrrole metabolism |
| GO:0006260 | DNA replication | tetrapyrrole metabolism |
| GO:0090305 | nucleic acid phosphodiester bond hydrolysis | tetrapyrrole metabolism |
| GO:0006396 | RNA processing | tetrapyrrole metabolism |
| GO:0008380 | RNA splicing | tetrapyrrole metabolism |
| GO:0006397 | mRNA processing | tetrapyrrole metabolism |
| GO:0072528 | pyrimidine-containing compound biosynthetic process | tetrapyrrole metabolism |
| GO:0072527 | pyrimidine-containing compound metabolic process | tetrapyrrole metabolism |
| GO:0072521 | purine-containing compound metabolic process | tetrapyrrole metabolism |
| GO:0034660 | ncRNA metabolic process | tetrapyrrole metabolism |
| GO:0043414 | macromolecule methylation | macromolecule methylation |
| GO:0018193 | peptidyl-amino acid modification | macromolecule methylation |
| GO:0031329 | regulation of cellular catabolic process | macromolecule methylation |
| GO:0009057 | macromolecule catabolic process | macromolecule methylation |
| GO:0070647 | protein modification by small protein conjugation or removal | macromolecule methylation |
| GO:0044270 | cellular nitrogen compound catabolic process | macromolecule methylation |
| GO:0030163 | protein catabolic process | macromolecule methylation |
| GO:0016567 | protein ubiquitination | macromolecule methylation |
| GO:0009894 | regulation of catabolic process | macromolecule methylation |
| GO:0044282 | small molecule catabolic process | macromolecule methylation |
| GO:1901361 | organic cyclic compound catabolic process | macromolecule methylation |
| GO:0046777 | protein autophosphorylation | macromolecule methylation |
| GO:0009451 | RNA modification | macromolecule methylation |
| GO:0030029 | actin filament-based process | actin filament-based process |
| GO:0032787 | monocarboxylic acid metabolic process | actin filament-based process |
| GO:0046394 | carboxylic acid biosynthetic process | actin filament-based process |
| GO:0051301 | cell division | actin filament-based process |
| GO:1901605 | alpha-amino acid metabolic process | actin filament-based process |
| GO:0009132 | nucleoside diphosphate metabolic process | actin filament-based process |
| GO:0008610 | lipid biosynthetic process | actin filament-based process |
| GO:0009141 | nucleoside triphosphate metabolic process | actin filament-based process |
| GO:0006720 | isoprenoid metabolic process | actin filament-based process |
| GO:0007049 | cell cycle | actin filament-based process |
| GO:0006643 | membrane lipid metabolic process | actin filament-based process |
| GO:0008219 | cell death | actin filament-based process |
| GO:1903047 | mitotic cell cycle process | actin filament-based process |
| GO:0006915 | apoptotic process | actin filament-based process |
| GO:0006091 | generation of precursor metabolites and energy | generation of precursor metabolites and energy |
| GO:0006732 | coenzyme metabolic process | coenzyme metabolism |
| GO:0006779 | porphyrin-containing compound biosynthetic process | coenzyme metabolism |
| GO:0044707 | single-multicellular organism process | single-multicellular organism process |
| GO:0072358 | cardiovascular system development | single-multicellular organism process |
| GO:0055123 | digestive system development | single-multicellular organism process |
| GO:0061008 | hepaticobiliary system development | single-multicellular organism process |
| GO:0001501 | skeletal system development | single-multicellular organism process |
| GO:0001889 | liver development | single-multicellular organism process |
| GO:0031016 | pancreas development | single-multicellular organism process |
| GO:0048732 | gland development | single-multicellular organism process |
| GO:0051186 | cofactor metabolic process | cofactor metabolism |

**Table S5.** Eleven genes previously described as candidates influencing craniofacial divergence are differentially expressed between generalists and specialists (McGirr and Martin 2017).

| scaffold | log_2_ FC | *P* | symbol | comparison | stage |
| --- | --- | --- | --- | --- | --- |
| 15151665 | 4.49 | <0.01 | znf664 | generalist vs. scale-eater | 17-20 dpf |
| 15150619 | 2.79 | <0.01 | abcg5 | generalist vs. scale-eater | 8-10 dpf |
| 15150999 | 1.22 | 0.04 | lrp1b | generalist vs. snail-eater | 17-20 dpf |
| 15151015 | 1.07 | 0.02 | gmds | generalist vs. scale-eater | 17-20 dpf |
| 15150619 | 0.91 | 0.01 | dync2li1 | generalist vs. scale-eater | 8-10 dpf |
| 15151665 | 0.7 | 0.04 | fam49b | generalist vs. scale-eater | 8-10 dpf |
| 15150480 | -0.51 | 0.01 | tmem30a | generalist vs. scale-eater | 8-10 dpf |
| 15150538 | -0.62 | 0.01 | fam172a | generalist vs. scale-eater | 8-10 dpf |
| 15151075 | -0.64 | <0.01 | atp8a1 | generalist vs. scale-eater | 8-10 dpf |
| 15150670 | -0.73 | 0.01 | ash1l | generalist vs. scale-eater | 8-10 dpf |
| 15150619 | -1.16 | 0.03 | hint1 | generalist vs. scale-eater | 17-20 dpf |

**Table S6.** 68 out of 84 gene regions containing fixed variants show signs of a hard sweep (estimated by SweeD; CLR > 95^th^ percentile across their respective scaffolds).

| scaffold | fixed SNPs | log2 fold change | adjusted *P* | stage | CLR | *Cyprinodon* gene symbol |
| --- | --- | --- | --- | --- | --- | --- |
| 15150501 | 1 | 0.68 | 0.03 | 8-10dpf | 0.42 | LOC107082156 |
| 15150501 | 1 | -1.13 | 0.03 | 17-20dpf | 0.42 | LOC107082156 |
| 15150501 | 4 | -1.26 | 0.00 | 8-10dpf | 0.42 | LOC107082264 |
| 15151439 | 2 | -0.62 | 0.02 | 8-10dpf | 0.42 | LOC107100553 |
| 15151189 | 10 | 0.84 | 0.00 | 8-10dpf | 0.39 | LOC107097945 |
| 15151015 | 1 | -1.23 | 0.03 | 8-10dpf | 0.36 | LOC107095655 |
| 15150556 | 1 | -0.63 | 0.03 | 8-10dpf | 0.33 | mef2c |
| 15150556 | 1 | -1.13 | 0.00 | 17-20dpf | 0.33 | mef2c |
| 15150680 | 1 | -1.25 | 0.00 | 8-10dpf | 0.31 | plgrkt |
| 15150776 | 1 | -1.02 | 0.03 | 17-20dpf | 0.30 | LOC107091063 |
| 15151452 | 1 | 0.74 | 0.00 | 8-10dpf | 0.28 | reck |
| 15150730 | 1 | 1.32 | 0.00 | 8-10dpf | 0.24 | exosc4 |
| 15151162 | 26 | -1.20 | 0.02 | 8-10dpf | 0.23 | LOC107097607 |
| 15151066 | 2 | 1.43 | 0.00 | 8-10dpf | 0.23 | dbf4 |
| 15151810 | 3 | -0.97 | 0.01 | 8-10dpf | 0.23 | LOC107103000 |
| 15151726 | 1 | 0.87 | 0.00 | 8-10dpf | 0.22 | LOC107102549 |
| 15150691 | 2 | 0.92 | 0.04 | 8-10dpf | 0.20 | LOC107089095 |
| 15150455 | 3 | 0.58 | 0.05 | 8-10dpf | 0.20 | fam188a |
| 15150455 | 1 | -1.30 | 0.00 | 8-10dpf | 0.20 | LOC107102995 |
| 15151892 | 1 | -1.34 | 0.01 | 17-20dpf | 0.19 | loxl3 |
| 15151892 | 1 | 1.02 | 0.01 | 8-10dpf | 0.19 | loxl3 |
| 15151400 | 2 | -1.48 | 0.00 | 8-10dpf | 0.18 | LOC107100233 |
| 15150688 | 2 | 0.62 | 0.01 | 8-10dpf | 0.18 | LOC107089013 |
| 15150854 | 2 | -1.13 | 0.00 | 8-10dpf | 0.18 | lmo7 |
| 15150495 | 1 | -1.35 | 0.01 | 8-10dpf | 0.18 | adgrg2 |
| 15150702 | 2 | -0.54 | 0.00 | 8-10dpf | 0.17 | cct8 |
| 15150702 | 3 | 0.69 | 0.03 | 8-10dpf | 0.17 | LOC107089362 |
| 15150702 | 15 | 1.73 | 0.00 | 8-10dpf | 0.17 | LOC107089382 |
| 15150702 | 15 | -2.32 | 0.00 | 17-20dpf | 0.17 | LOC107089382 |
| 15150924 | 1 | -0.92 | 0.00 | 8-10dpf | 0.17 | LOC107094239 |
| 15150533 | 1 | 1.11 | 0.02 | 8-10dpf | 0.16 | erap2 |
| 15150763 | 1 | -2.68 | 0.00 | 8-10dpf | 0.16 | LOC107090753 |
| 15150467 | 3 | -0.79 | 0.04 | 17-20dpf | 0.15 | nxn |
| 15151167 | 19 | 1.34 | 0.00 | 17-20dpf | 0.15 | LOC107097675 |
| 15151665 | 2 | 0.70 | 0.04 | 8-10dpf | 0.15 | fam49b |
| 15151665 | 6 | 4.49 | 0.00 | 17-20dpf | 0.15 | znf664 |
| 15150634 | 1 | 0.61 | 0.02 | 8-10dpf | 0.15 | xpo4 |
| 15151075 | 7 | -0.64 | 0.00 | 8-10dpf | 0.15 | atp8a1 |
| 15151905 | 1 | 0.61 | 0.02 | 8-10dpf | 0.14 | LOC107103455 |
| 15151905 | 1 | 0.88 | 0.03 | 8-10dpf | 0.14 | pxmp4 |
| 15150457 | 5 | -0.64 | 0.00 | 8-10dpf | 0.12 | ppp1r13b |
| 15150457 | 5 | 0.96 | 0.00 | 17-20dpf | 0.12 | ppp1r13b |
| 15152211 | 5 | 0.68 | 0.04 | 17-20dpf | 0.12 | atf6b |
| 15150651 | 2 | -2.40 | 0.00 | 17-20dpf | 0.12 | LOC107087896 |
| 15150673 | 4 | -0.86 | 0.00 | 8-10dpf | 0.12 | papd5 |
| 15151409 | 2 | -1.00 | 0.04 | 8-10dpf | 0.12 | LOC107100292 |
| 15150711 | 10 | -5.42 | 0.00 | 8-10dpf | 0.12 | fbxo32 |
| 15150711 | 8 | -6.16 | 0.00 | 8-10dpf | 0.12 | klhl38 |
| 15150599 | 1 | 0.66 | 0.04 | 8-10dpf | 0.11 | snx29 |
| 15150825 | 1 | 4.68 | 0.00 | 8-10dpf | 0.11 | pkd1l1 |
| 15150825 | 1 | 1.34 | 0.00 | 8-10dpf | 0.11 | skida1 |
| 15150487 | 2 | -0.83 | 0.00 | 8-10dpf | 0.10 | st7l |
| 15150621 | 20 | -1.51 | 0.01 | 17-20dpf | 0.09 | kcnab1 |
| 15150536 | 40 | 0.66 | 0.01 | 8-10dpf | 0.09 | eif2b3 |
| 15150536 | 1 | -0.96 | 0.04 | 8-10dpf | 0.09 | LOC107083768 |
| 15150536 | 18 | -1.04 | 0.02 | 8-10dpf | 0.09 | plk3 |
| 15150548 | 2 | -0.56 | 0.05 | 17-20dpf | 0.09 | LOC107084243 |
| 15150538 | 1 | -0.62 | 0.01 | 8-10dpf | 0.09 | fam172a |
| 15150538 | 2 | 0.73 | 0.05 | 17-20dpf | 0.09 | rtkn |
| 15150538 | 2 | -0.87 | 0.01 | 8-10dpf | 0.09 | rtkn |
| 15150453 | 3 | -1.73 | 0.00 | 8-10dpf | 0.08 | LOC107084596 |
| 15150453 | 3 | -0.85 | 0.05 | 17-20dpf | 0.08 | LOC107084596 |
| 15150453 | 3 | -0.59 | 0.01 | 8-10dpf | 0.08 | LOC107084689 |
| 15151058 | 1 | 1.34 | 0.02 | 8-10dpf | 0.08 | LOC107096196 |
| 15150508 | 1 | -0.72 | 0.04 | 17-20dpf | 0.07 | atic |
| 15150508 | 1 | 0.65 | 0.01 | 8-10dpf | 0.07 | atic |
| 15150480 | 1 | -0.51 | 0.01 | 8-10dpf | 0.07 | tmem30a |
| 15150463 | 1 | -0.66 | 0.05 | 17-20dpf | 0.07 | stx5 |
| 15151111 | 1 | -2.18 | 0.00 | 8-10dpf | 0.07 | LOC107096914 |
| 15151111 | 1 | -3.83 | 0.00 | 17-20dpf | 0.07 | LOC107096914 |
| 15151111 | 1 | -1.55 | 0.00 | 8-10dpf | 0.07 | LOC107096921 |
| 15150652 | 6 | 0.71 | 0.05 | 8-10dpf | 0.07 | LOC107087924 |
| 15151119 | 21 | -2.06 | 0.00 | 17-20dpf | 0.06 | LOC107097014 |
| 15151119 | 21 | -0.92 | 0.03 | 8-10dpf | 0.06 | LOC107097014 |
| 15151119 | 22 | -0.92 | 0.00 | 8-10dpf | 0.06 | LOC107097016 |
| 15150922 | 2 | -0.78 | 0.02 | 17-20dpf | 0.06 | LOC107094191 |


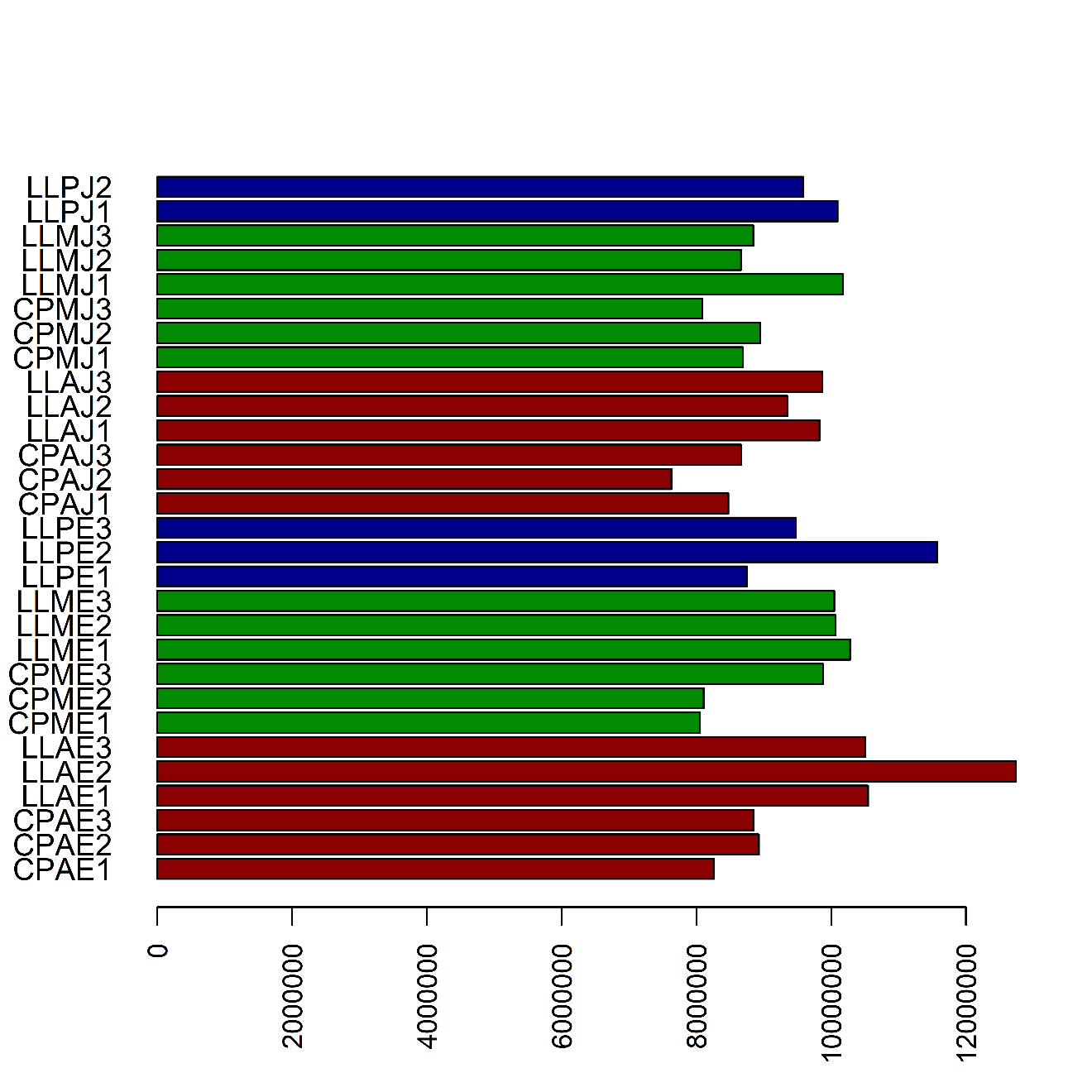


reads mapped to annotated features

**Fig. S1.** A similar number of reads map to annotated features across generalists (red), snail-eaters (green), and scale-eaters (blue) (ANOVA; 8-10 dpf *p* = 0.47; 17-20 dpf *p* = 0.33). CP = Crescent Pond, LL = Little Lake, E = 8-10 dpf, J = 17-20 dpf).


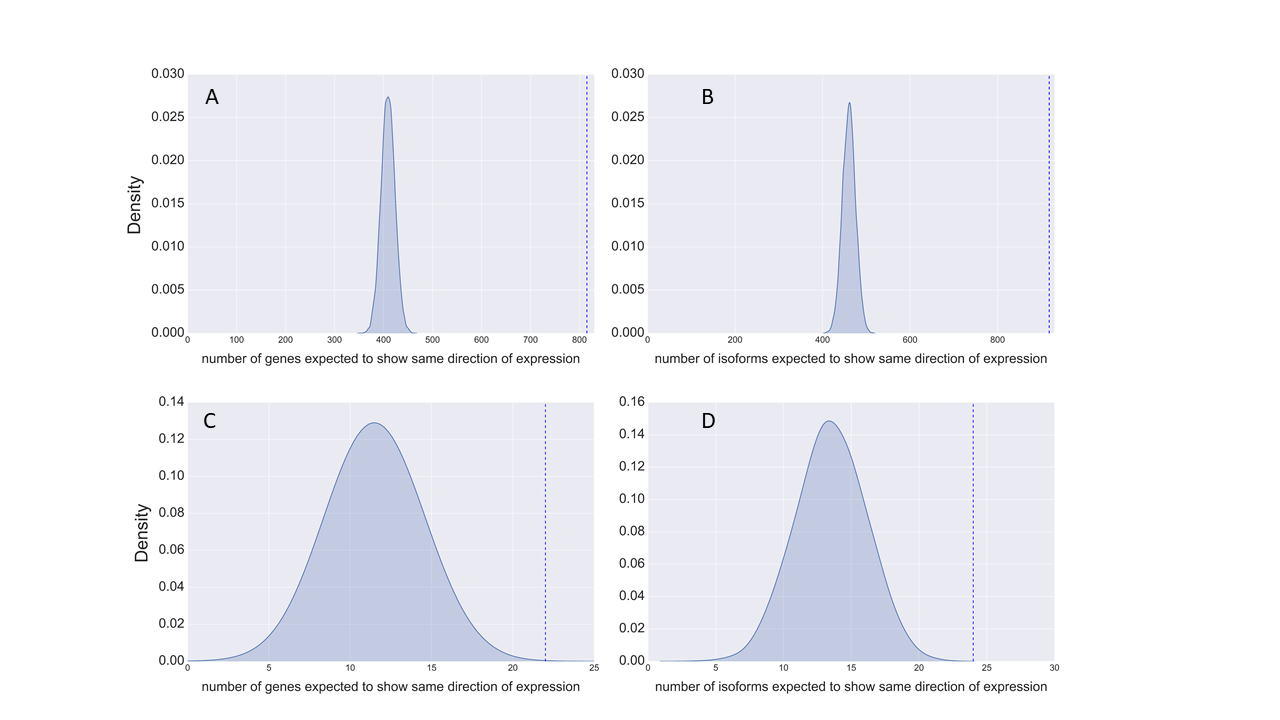


**Fig. S2. Null distributions of parallel changes in gene expression between specialists.** Kernel density plots show the null distribution for the number of genes (A and C) and isoforms (B and D) expected to show the same direction of expression in specialists relative to generalists. We performed 10,000 permutations sampling from a binomial distribution to estimate the expected number of genes and isoforms showing shared expression. The actual number of genes and isoforms showing shared directions of expression are indicated by blue dotted lines. A and B show distributions for gene and isoform expression at 8-10 dpf. C and D show distributions for gene and isoform expression at 17-20 dpf. Significantly more genes and isoforms show the same expression pattern in specialists relative to generalists at both developmental time points (*P <* 1.0 × 10^-4^).


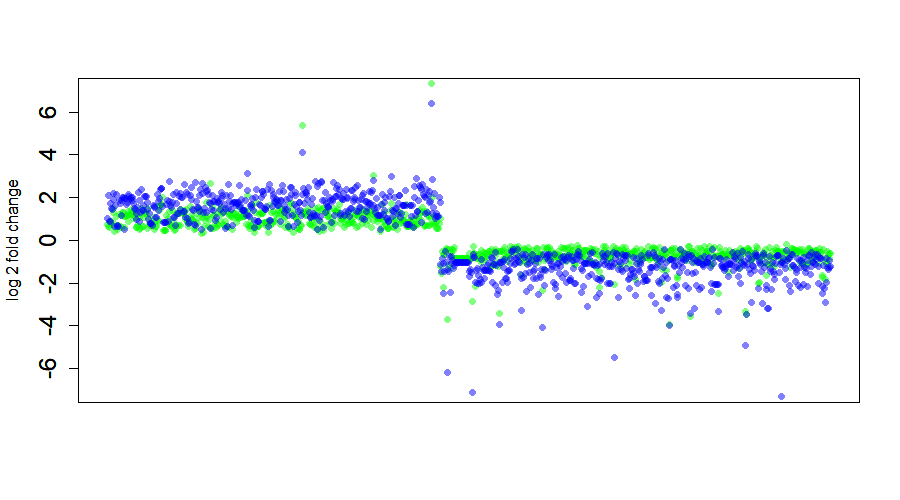


isoforms showing the same direction of expression in specialists

**Fig. S3. Parallel changes in isoform expression between specialists at 8-10 dpf.** 497 differentially expressed isoforms showed lower expression in both specialist species compared to generalists, while 424 showed higher expression in specialists. Blue points indicate log_2_ fold change for genes differentially expressed between generalists and scale-eaters and green shows log_2_ fold change for genes differentially expressed between generalists and snail-eaters.


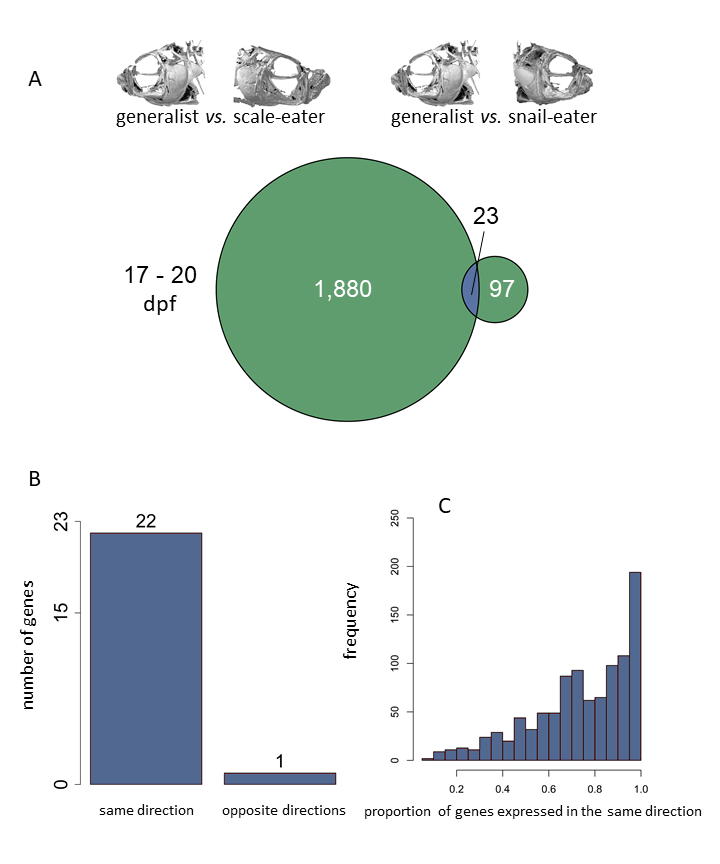


**Fig. S4. Significant parallel evolution of gene expression between specialists despite divergent trophic adaptation.** Circles illustrate genes differentially expressed in 17-20 dpf whole-body tissue for generalists *vs.* scale-eaters (left) and generalists *vs.* snail-eaters (right). Genes showing differential expression in both comparisons are shown in blue, and those showing divergent expression patterns unique to each specialist are green. Significantly more genes show differential expression in both comparisons than expected by chance (Fisher’s exact test, *P* < 1.0 × 10^-16^). B) Significantly more genes show the same direction of expression in specialists relative to generalists than expected by chance (10,000 permutations; *P* < 1.0 × 10^-4^; Fig. S2). C) Distribution of the proportion of genes differentially expressed in the same direction between specialists relative to generalists after 1,000 down sampling permutations where groups of generalists and snail-eaters were randomly sampled to match scale-eater sample sizes (n = 2) show that parallel expression is robust to variation in sample size (median number of genes common to both comparisons = 16).


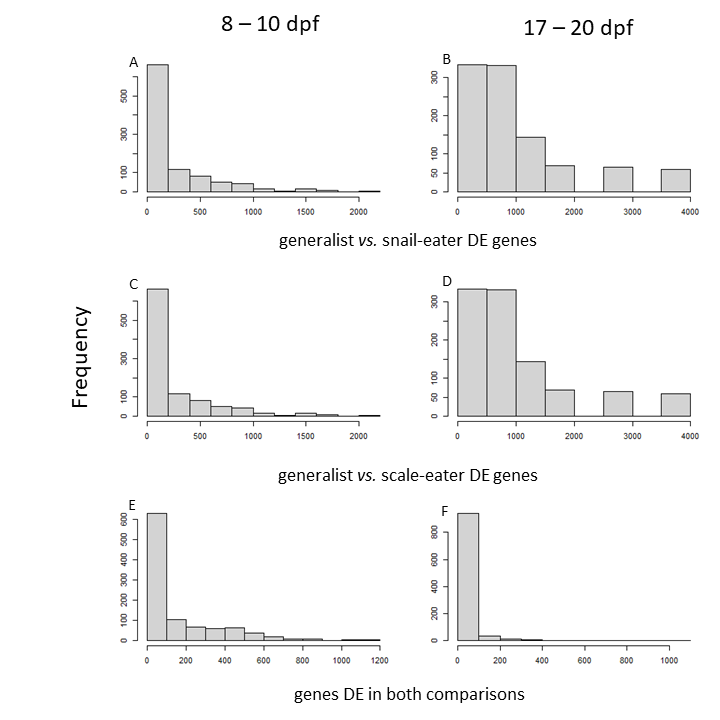


**Fig. S5. Down sampling permutations.** Distribution of genes differentially expressed (DE) between generalists and snail-eaters (A and B), generalists and scale-eaters (C, and D), and genes DE in both comparisons (E and F) for 8-10 dpf (left) and 17-20 dpf (right) samples after 1000 down sampling permutations where groups of generalists and snail-eaters were randomly sampled to match scale-eater sample sizes (8-10 dpf, n = 3; 17-20 dpf, n = 2).


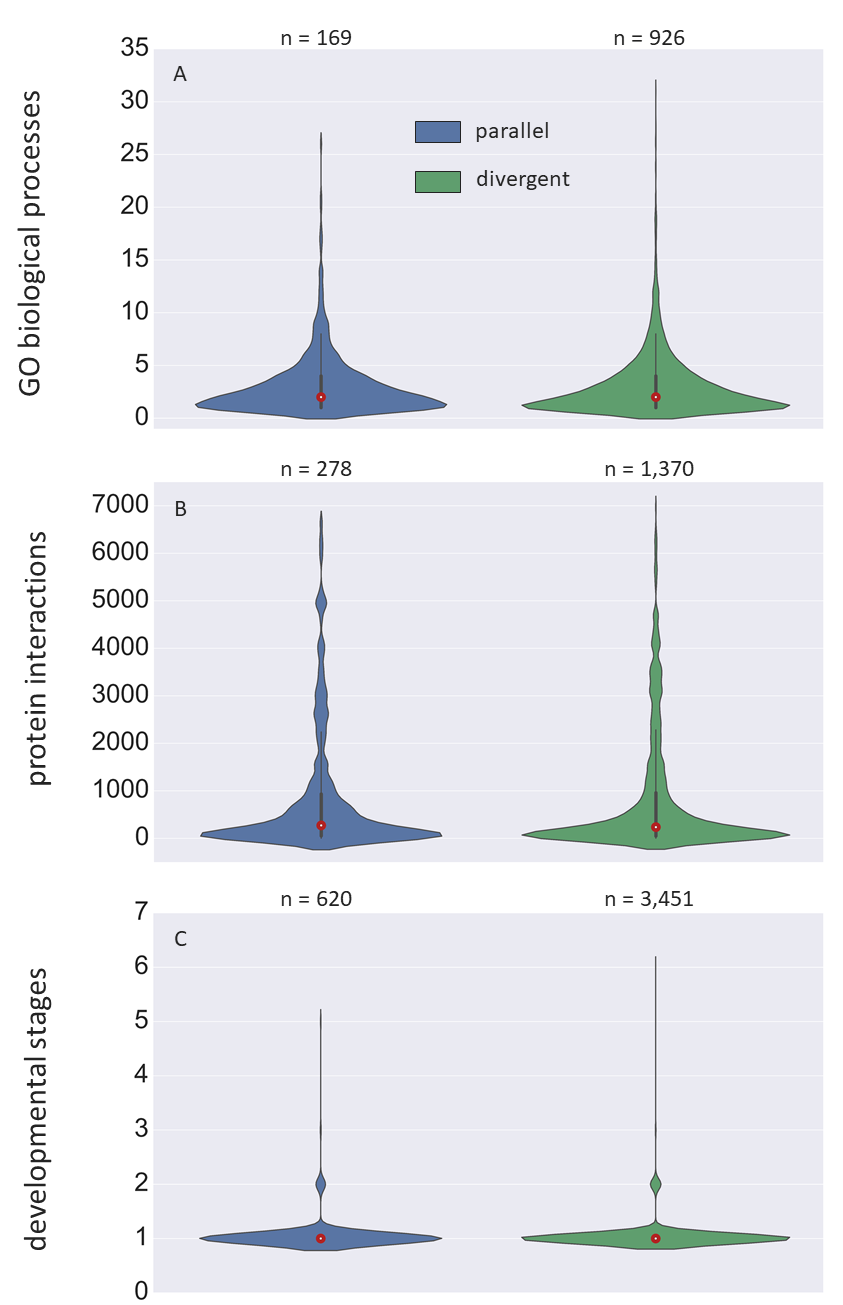


**Fig. S6. Genes showing parallel expression patterns in specialists are not more pleiotropic than genes showing divergent expression.** Violin plots show the distribution of pleiotropy estimates (GO biological processes (A), protein-protein interactions (B), and developmental stages expressed (C)) for genes showing parallel changes in expression (blue) and divergent changes in expression (green) between specialists relative to generalists at 8-10 dpf. Red dots show the median, thick black bars show interquartile ranges and thin bars show 95% confidence intervals. Genes showing parallel expression are not significantly more or less pleiotropic than divergently expressed genes (GLM; biological processes: *P* = 0.67; PPIs: *P* = 0.09; developmental stages: *P* = 0.89).


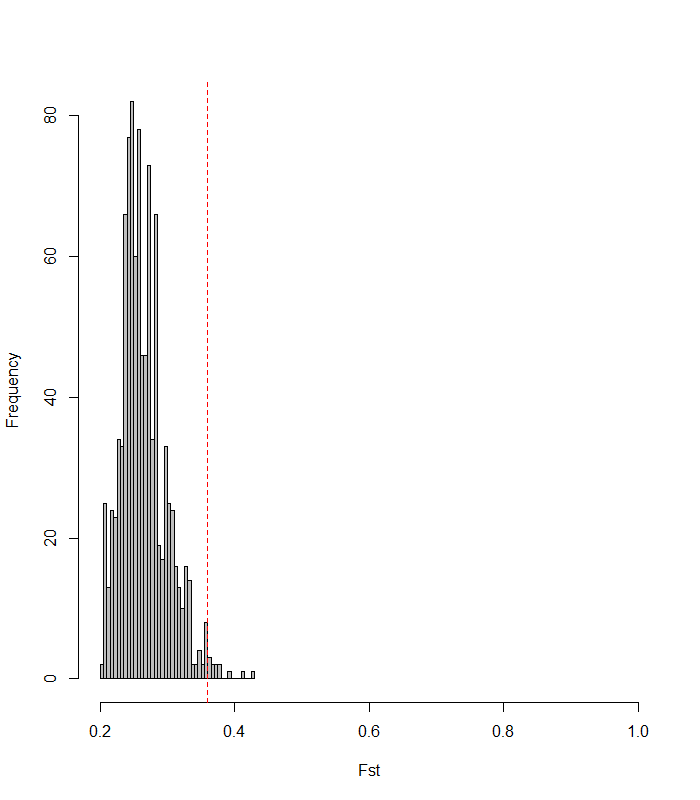

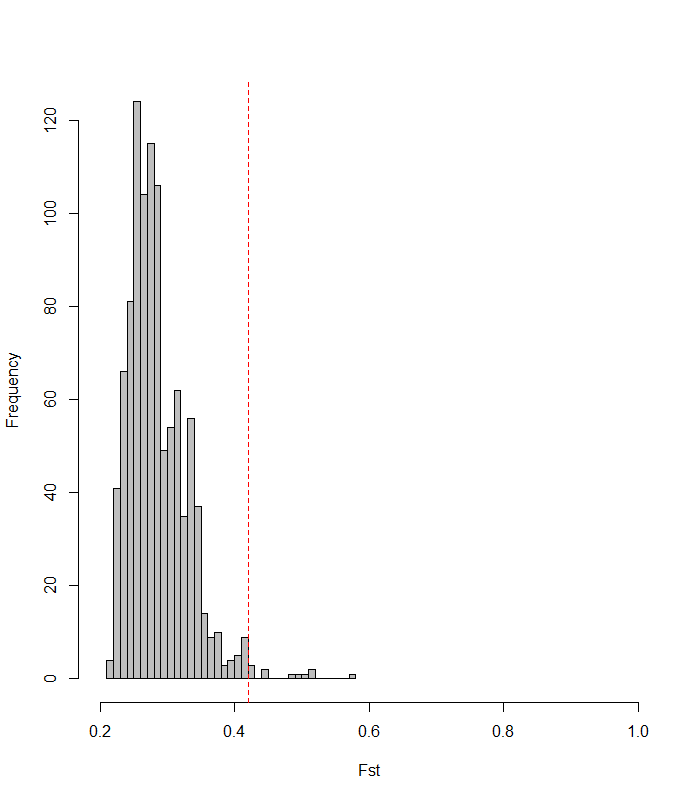


B

A

**Fig. S7. F_st_ permutations to determine significantly differentiated SNPs.** We performed 1,000 permutations calculating genome-wide F_st_ between randomly subsampled groups in order to identify non-randomly differentiated genomic regions between species A) 99^th^ percentile estimates of F_st_ across all SNPs between randomly sampled generalists and snail-eaters (n = 13 vs. n = 11). B) 99^th^ percentile estimates of F_st_ across all SNPs between randomly sampled generalists and scale-eaters (n = 13 vs. n = 9). We took the 99th percentile of these distributions to set a threshold defining significantly high divergence (red dotted lines; F_st_ > 0.36 for generalists *vs*. snail-eaters; F_st_ > 0.42 for generalists *vs.* scale-eaters).

**Coding Supplement**

#### Genome-Wide Variation Pipeline ####

# HARD FILTER SNPS

java -jar GenomeAnalysisTK.jar -T VariantFiltration -R Cyprinodon.fasta -V raw_snps.vcf --filterExpression "QD < 2.0 || FS > 60.0 || MQ < 40.0 || MQRankSum < -12.5 || ReadPosRankSum < -8.0" --filterName "my_snp_filter" -o snps.vcf

vcftools --vcf snps.vcf --maf 0.05 --max-missing 0.9 --recode --out filtered_snps

# Estimate Fst

vcftools --vcf filtered_snps.vcf --out scale_v_gen_fst --weir-fst-pop sclae_eaters_indivs.txt --weir-fst-pop generalists_indivs.txt"

#### RNA-seq Pipeline ####

# Trim fastq feads

trim_galore -q 20 --paired --illumina CPAE1-2nd_Prep_CGCTCATT-GTACTGAC_S217_L005_R1_001.fastq CPAE1-2nd_Prep_CGCTCATT-GTACTGAC_S217_L005_R2_001.fastq

# Deduplicate

java -jar picard.jar MarkDuplicates INPUT=axmE1.sort.bam OUTPUT=axmE1.sort.dedup.bam METRICS_FILE=axmE1.metrics.txt MAX_FILE_HANDLES=1000

# Create GTF file from GFF3

gffread ref_C_variegatus-1.0_scaffolds.gff3 -T -o my.gtf

# STAR (mapping reads)

module load star

module load subread

# CREATE GENOME INDEXES

star --runThreadN 4 \

--runMode genomeGenerate \

--genomeDir /proj/cmarlab/users/joe/outgroups/rna/genome_dir \

--genomeFastaFiles /proj/cmarlab/users/joe/outgroups/rna/Cyprinodon_NW.fasta \

--sjdbGTFfile ref_C_variegatus-1.0_scaffolds.gff3 \

--sjdbGTFtagExonParentTranscript Parent \

--sjdbOverhang 149

#MAP READS

star --runThreadN 4 \

--genomeDir /proj/cmarlab/users/joe/outgroups/rna/genome_dir \

--readFilesIn LLAJ2-2nd_Prep_ATTACTCG-ATAGAGGC_S226_L005_R1_001_val_1.fq LLAJ2-2nd_Prep_ATTACTCG-ATAGAGGC_S226_L005_R2_001_val_2.fq \

--outFileNamePrefix LLAJ2

# Count Reads

featureCounts -p -a my.gtf.geneid -B -C -G Cyprinodon_NW.fasta -s 2 -T 4 -o counts_geneid CPAE1.rna.bam CPAE2.rna.bam CPAE3.rna.bam CPAJ1.rna.bam CPAJ2.rna.bam CPAJ3.rna.bam CPME1.rna.bam CPME2.rna.bam CPME3.rna.bam CPMJ1.rna.bam CPMJ2.rna.bam CPMJ3.rna.bam CPPE1.rna.bam CPPE2.rna.bam CPPE3.rna.bam CPPJ1.rna.bam CPPJ2.rna.bam LLAE1.rna.bam LLAE2.rna.bam LLAE3.rna.bam LLAJ1.rna.bam LLAJ2.rna.bam LLAJ3.rna.bam LLME1.rna.bam LLME2.rna.bam LLME3.rna.bam LLMJ1.rna.bam LLMJ2.rna.bam LLMJ3.rna.bam

# Differential expression in R using DESeq2

cts <- as.matrix(read.table(cts_data ,sep = "\t",header = TRUE,row.names=1))

colData <- as.matrix(read.table(col_data ,header = TRUE,row.names=1))

comp <- "species"

dds <- DESeqDataSetFromMatrix(countData = cts, colData = colData, design= ~ species)

unused <- dds[ rowSums(counts(dds)) < 1, ]

dds <- dds[ rowSums(counts(dds)) > 1, ]

dds <- DESeq(dds)

res <- results(dds, independentFiltering=FALSE)

res$pvalue[res$baseMean < 10] <- NA

res$padj <- p.adjust(res$pvalue, method="BH")

res <- results(dds, alpha=0.05)

resOrdered <- res[order(res$padj),]

summary(res)

resLFC <- lfcShrink(dds, coef=2, res=res)

res_ordered <- as.data.frame(resOrdered)

write.csv(res_ordered, file= cts_data_genes)
